# Improving Diabetic Foot Care Through a Multidisciplinary Clinic: Experience From Pilgrim Hospital, UK

**DOI:** 10.7759/cureus.95480

**Published:** 2025-10-27

**Authors:** Mahir Yousuff, Nyi Htwe, Ahmad Joumah, Jeeno Jayan, Nijil Vasukutty

**Affiliations:** 1 Trauma and Orthopaedics, King’s Mill Hospital, Mansfield, GBR; 2 Trauma and Orthopaedics, Pilgrim Hospital, Boston, GBR; 3 Diabetes and Endocrinology, Pilgrim Hospital, Boston, GBR

**Keywords:** charcot foot, diabetic foot ulcer (dfu), lower limb amputation, management of diabetic foot, multidisciplinary clinic

## Abstract

Background

Diabetic foot ulcers are a major cause of morbidity and mortality in people with diabetes. Although multidisciplinary team (MDT) models are increasingly recognised as best practice, they remain under-implemented across the United Kingdom. This study evaluated the clinical impact of introducing a diabetic foot MDT service at a district general hospital in the United Kingdom.

Methodology

This retrospective, cohort study compared clinical outcomes in two patient groups, namely, those managed before (Cycle 1; January to June 2016, n = 72 patients, 85 lesions) and after (Cycle 2; January to June 2019, n = 90 patients, 111 lesions) the implementation of the MDT. Primary outcomes included major amputation and mortality, while secondary outcomes included healing rate. Further data were collected on demographics, interventions, service outcomes, and Charcot foot.

Results

Following MDT implementation, major amputations decreased from 11% to 6% (p = 0.196), three-year mortality reduced from 39% to 29% (p = 0.180), and healing rate improved from 64% to 74% (p = 0.289), all clinically meaningful although not statistically significant. Ulcer healing time improved significantly from 300 to 244 days (p = 0.0375), and readmission rates due to recurrent or new lesions dropped from 47% to 22% (p = 0.0008). All post-MDT patients received vascular review, with an increase in revascularisation and orthopaedic surgeries. Charcot foot cases demonstrated stability post-treatment, with no reactivations over three years.

Conclusions

Implementation of a diabetic foot MDT significantly improved healing time and reduced readmissions, with clinically meaningful reductions in amputation and mortality. These findings support the adoption of integrated MDT models in district general hospitals to optimise diabetic foot outcomes in the United Kingdom.

## Introduction

Diabetic foot ulcers (DFUs) represent a significant and growing burden in the United Kingdom, accounting for nearly 1% of the National Health Service (NHS) budget and costing more than breast, prostate, and lung cancer combined [1]. The lifetime risk of a person with diabetes developing a foot ulcer is approximately 19-34% [2]. DFUs typically arise from peripheral neuropathy, peripheral arterial disease, and structural foot abnormalities. Without treatment, these ulcers can progress to tissue necrosis and infection, including cellulitis and osteomyelitis, which may ultimately lead to amputation or death [3]. As the causes are multifactorial, guidelines from National Institute for Health and Care Excellence [4] and International Working Group on the Diabetic Foot [5] recommend, as the gold standard, a multidisciplinary team (MDT) bringing together specialists in diabetes, podiatry, vascular surgery, orthopaedic surgery, microbiology, and wound care to allow interventions in areas such as glycaemic control, offloading, early detection of infection to debridement, and revascularisation surgery. Ever since the establishment of the first ever diabetic foot MDT at King’s College London in 1981 [6], these MDTs have been studied in the literature with systematic reviews and meta-analyses [3,7], reporting a decrease in ulcer healing time, amputation rate, and mortality rate. Despite the success of these MDTs, reportedly one in six hospitals in the United Kingdom has no dedicated multidisciplinary foot team [8]. Hence, due to variation in MDT composition [9] and patient characteristics, local studies are needed [10,11].

The clinic at Pilgrim Hospital, Boston, UK, is a district general hospital which works within the United Lincolnshire Hospitals Trust to serve a population of nearly 700,000 people. Before 2018, diabetic foot care in the hospital was fragmented. Patients attending the diabetic foot clinic were typically reviewed only by a diabetologist or podiatrist. Any need for surgical or vascular input required a separate referral, often resulting in delayed assessments and disjointed care. In response to these service gaps, a new one-stop MDT clinic was introduced in 2018. This service integrated permanent vascular surgical support alongside regular orthopaedic input, enabling comprehensive, same-day assessment and management for complex diabetic foot cases. This study evaluates the clinical and operational impact of the one-stop MDT clinic on diabetic foot outcomes at our hospital, comparing data from before and after its implementation.

## Materials and methods

A two-cycle retrospective audit was conducted to evaluate the impact of implementing a one-stop MDT diabetic foot clinic at Pilgrim Hospital. Data were collected during two six-month periods, namely, before the creation of the MDT clinic (Cycle 1; 1st January 2016 to 30th June 2016) and after its introduction (Cycle 2; 1st January 2019 to 30th June 2019). This audit was registered with the local clinical governance department (approval number: P0512) and complied with institutional standards for service evaluation. Ethical approval was not required.

Clinic details

The clinic operated during weekdays in the outpatient setting. Patients were referred to the diabetic foot MDT clinic primarily from community podiatry services, with additional referrals from general practitioners (GPs) and patients discharged from secondary care following an inpatient stay. Referrals were then triaged by a diabetes physician.

Each clinic session included a diabetes physician, tissue viability nurse specialist, podiatrist, and vascular surgeon. The vascular surgeon would assess all patients for lower limb perfusion, including measurement of the ankle-brachial pressure index. Revascularisation or amputation procedures were considered if evidence of critical limb ischaemia or deteriorating ulcers. An orthopaedic surgeon attended on scheduled days to review patients for osteomyelitis, Charcot foot, or any ulcer requiring debridement.

Data collection

All consecutive patients attending the diabetic foot clinic during each six-month audit period were identified from electronic clinic lists. Patients were then screened for eligibility according to inclusion and exclusion criteria. Patient information was obtained retrospectively from electronic medical records and clinic documentation. Baseline demographic and clinical characteristics, as well as details of interventions, were recorded. The primary outcomes were major amputation and three-year mortality. Secondary outcomes included healing rate, with further data collected on time to healing, need for vascular and orthopaedic review, revascularisation procedures, minor amputations, and readmission to the MDT clinic. Data were also collected on Charcot foot management and outcomes. All patients were followed for three years after their index clinic attendance.

Inclusion and exclusion criteria

**Figure 1:**
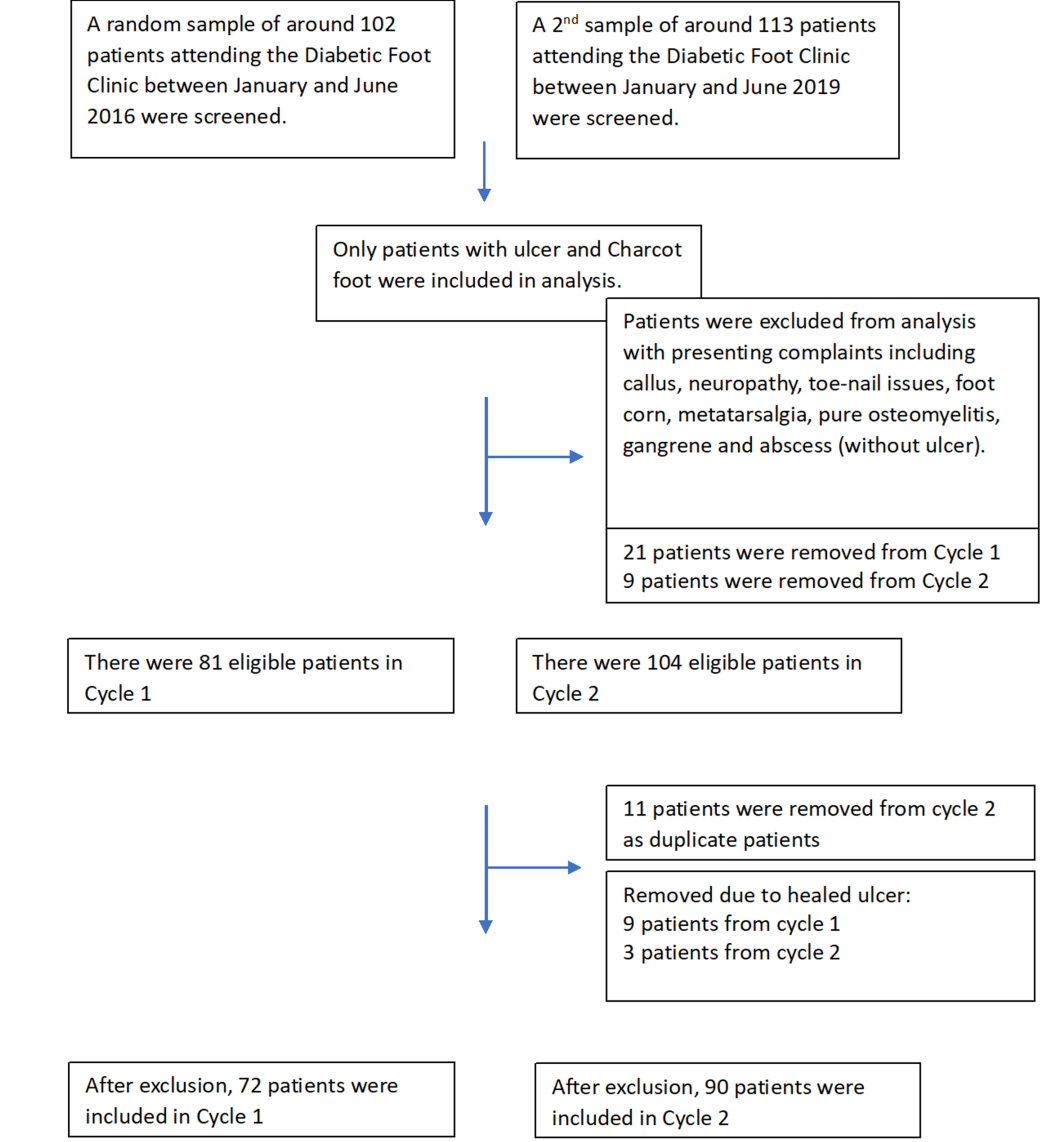
Patient inclusion and exclusion flow diagram. Flow of patients screened, excluded, and included across two audit cycles. Exclusions: non-ulcerative complaints, isolated osteomyelitis, gangrene, abscess without ulcer, duplicates, or healed ulcers at first visit.

Patients were eligible if they were aged 18 years or older, had a diagnosis of either DFU or Charcot foot, and were reviewed in the diabetic foot clinic during one of the two study periods. Patients were excluded if they presented with isolated callus, metatarsalgia, isolated neuropathy, or non-ulcerative osteomyelitis or gangrene. Patients who had a healed lesion at their first clinic visit and were discharged immediately were also excluded. Finally, duplicated patients from Cycle 1 were excluded from Cycle 2 analysis. The inclusion and exclusion process is summarised in Figure 1 and Table 1.

**Table 1:** Final patient cohort after applying exclusion criteria. Patient cohort through the study during two six-month audit cycles. Patients were excluded if they presented with non-ulcerative lesions (callus, metatarsalgia, isolated neuropathy, non-ulcerative osteomyelitis, or gangrene), duplicate presentations across cycles, or healed lesions at first visit with immediate discharge. Values represent patient counts unless otherwise stated. “Lesions” refer to either Charcot foot, ulcer, or non-ulcerative lesions mentioned above; “Charcot” refers to Charcot neuroarthropathy; “Ulcer” refers to diabetic foot ulcer.

	Cycle 1 (2016)	Cycle 2 (2019)
Patients	102	113
Patients excluded based on lesion	21	9
Patients with Charcot or ulcer	81	104
Total lesions	94	130
Duplicate patients across cycles	-	11
Duplicate lesions across cycles	-	16
Patients discharged on day 1	9	3
Patients after exclusion	72	90
Lesions after exclusion	85	111
Ulcer	73	101
Charcot	12	10

Definitions

A “diabetic foot ulcer (DFU)” was defined as a break in the skin involving the epidermis and at least part of the dermis. The “index lesion” referred to the ulcer that was the primary focus of treatment at enrolment. Ulcers could include associated cellulitis, osteomyelitis, or abscess. “Charcot foot” was defined as a progressive neuroarthropathy occurring in the context of diabetic peripheral neuropathy, characterised by inflammation, bone fragmentation, and deformity.

“Major amputation” was defined as any amputation through or proximal to the ankle joint, including below-knee amputation or above-knee amputation. “Minor amputation” referred to procedures distal to the ankle joint, including toe, ray, or trans-metatarsal amputations. A “healed” ulcer was one that had completely epithelialised, as recorded in clinic documentation. Patients were considered “not healed” if the ulcer remained open, the patient was still under MDT review, or the patient had died before healing occurred.

Mortality at three years was defined as death within three years of attending the MDT clinic with a new ulcer, regardless of whether it was the patient’s first ulcer.

Statistical analysis

Descriptive statistics were used to summarise patient demographics and clinical characteristics. Univariate analysis was performed using GraphPad Prism version 9, with comparisons made between pre- and post-MDT groups. Continuous variables were analysed using the Mann-Whitney U test. Categorical variables were compared using the chi-square test. A p-value <0.05 was considered statistically significant. Confidence intervals were not included as the audit focused on comparisons rather than precise effect estimation. Unless otherwise specified, outcomes are reported for the combined cohort of patients with DFU and Charcot foot, as both were managed within the same MDT pathway.

## Results

During the six-month period from 1st January 2016 to 30th June 2016 (Cycle 1), 102 patients were selected from a clinic list. In total, 21 patients were excluded based on lesion type, with a further nine excluded after presenting with a healed lesion. This was compared with the six-month period of 1st January 2019 to 30th June 2019 (Cycle 2), where 113 patients were selected, with nine excluded based on lesion type, three presenting with a healed lesion, and a further 11 patients with 16 lesions who were also part of Cycle 1. This left 72 patients with 85 lesions (73 ulcers and 12 Charcot foot) fulfilling the inclusion criteria for Cycle 1, and 90 patients with 111 lesions (101 ulcers and 10 Charcot foot) fulfilling the inclusion criteria for Cycle 2.

The baseline characteristics of the cohort are shown in Table 2. The two cohorts were similar, as noted by non-significant p-values. There were 60 ulcer and 12 Charcot patients in Cycle 1, compared to 80 and 10, respectively, in Cycle 2. Most people had a single lesion (82% and 77%), with the remainder of patients having two or more ulcers. The average age was 69 and 71 years old, respectively, with a male predominance (68% and 74%). Overall, 93% had type 2 diabetes in both cohorts, with similar HbA1c of 74.2 mmol/mol and 75.4 mmol/mol, respectively. There was no significant difference in smokers.

**Table 2:** Baseline characteristics and interventions. Comparison of baseline characteristics and interventions between patients in Cycle 1 (2016) and Cycle 2 (2019). Values are shown as n (%) unless otherwise indicated. All percentages are expressed to two significant figures. *: P-values <0.05 are considered statistically significant. HbA1c = glycated haemoglobin

	Cycle 1 (2016)	Cycle 2 (2019)	P-value
Baseline characteristics	(n = 72)	(n = 90)	
Patients with an ulcer	60 (83%)	80 (89%)	0.305
Patients with Charcot foot	12 (17%)	10 (11%)	
Patients with 1 lesion	59 (82%)	69 (77%)	0.412
Patients with >1 lesion	13 (18%)	21 (23%)	
Average age (years)	69	71	0.155
Male sex	49 (68%)	67 (74%)	0.370
Type 1 diabetes	5 (7%)	6 (7%)	0.944
Type 2 diabetes	67 (93%)	84 (93%)	
HbA1c (mmol/mol)	74.2	75.4	0.314
Active smoking	16 (22%)	25 (28%)	0.539
Ex-smokers	22 (31%)	30 (33%)	
Interventions			
Orthopaedic review	10 (14%)	19 (21%)	0.233
Orthopaedic surgery	2 (3%)	7 (8%)	0.167
Vascular review	42 (58%)	90 (100%)	0.0001*
Revascularisation surgery	6 (8%)	15 (17%)	0.117
Minor amputation	6 (8%)	9 (10%)	0.716
Major amputation	8 (11%)	5 (6%)	0.196

Following the introduction of the MDT, every patient in Cycle 2 received a vascular review, which was a significant improvement (58% vs. 100%, p = 0.0001). There were increases in orthopaedic reviews (14% to 21%) and surgery (3% to 8%); however, these did not reach statistical significance. There was an increase in revascularisation surgery (8% to 17%) and a slight increase in minor amputations (8% to 10%).

In terms of primary outcomes (Table 3), following MDT implementation, both the major amputation rate (11% to 6%) and three-year mortality rate (39% to 29%) fell, although neither met statistical significance (p = 0.196 and p = 0.180, respectively). In terms of secondary outcomes, 74% of lesions healed in Cycle 2 compared to 64% in Cycle 1, which was not statistically significant (p = 0.289).

**Table 3:** Primary and secondary outcomes before and after MDT implementation. Comparison of primary and secondary outcomes between Cycle 1 (2016) and Cycle 2 (2019). Primary outcomes (major amputation and mortality) include both ulcer and Charcot patients, reported per patient. Healing rate analysis is based on lesions (ulcer and Charcot combined). Values are shown as n (%). All percentages are expressed to two significant figures unless otherwise stated. P-values <0.05 are considered statistically significant MDT = multidisciplinary team

	Cycle 1 (2016)	Cycle 2 (2019)	P-value
Primary outcomes			
Major amputation	8 (11%)	5 (6%)	0.196
Mortality at 3 years	28 (39%)	26 (29%)	0.180
Secondary outcome: healing rate	(n = 85)	(n = 111)	0.289
Healed	54 (64%)	82 (74%)	
Not healed	17 (20%)	15 (14%)	
Amputation (minor and major)	14 (16%)	14 (13%)	

Service outcomes are available in Table 4. The time it took for a single ulcer (i.e., not Charcot foot) to heal reduced by 56 days post-MDT implementation, from 300 days to 244 days (p = 0.0375). However, once the index lesion healed, the number of people who stayed within the MDT (often due to developing another lesion) was 16 (22%) patients, spending 644 days (or 344 extra days on average) in Cycle 1 and 26 (29%) patients, spending 504 days (260 extra days on average) in Cycle 2. There was no significant difference between either patients who stayed extra (p = 0.336) or duration of time spent (p = 0.219). There was a significant reduction in patients readmitted to the MDT within three years from 47% to 22% (p = 0.0008). Of the 34 that were readmitted in Cycle 1, 20 were due to a new lesion and 14 due to a flare of a recurrent lesion. Of the 20 readmitted in Cycle 2, 11 and 9 were new and recurrent, respectively. The average number of days patients spent between discharge and subsequent readmission into the MDT was 499 in Cycle 1 and 364 in Cycle 2, showing no significant difference.

**Table 4:** Service outcomes. Service outcomes comparing Cycle 1 (2016) and Cycle 2 (2019). The index ulcer healing duration is reported for ulcer patients only. All other outcomes (remaining in MDT after healing, duration in MDT care, readmissions, and time to readmission) include both ulcer and Charcot patients, reported per patient. Values are shown as mean or n (%). All percentages are expressed to two significant figures. *: P-values <0.05 are considered statistically significant. MDT = multidisciplinary team

	Cycle 1 (2016)	Cycle 2 (2019)	P-value
Service outcomes			
Mean index ulcer healed duration	300 days	244 days	0.0375*
Patients remaining in MDT care after the index lesion healed	16 (22%)	26 (29%)	0.336
Mean duration in MDT care after the index lesion healed	644 days	504 days	0.219
Patients readmitted to MDT within 3 years	34 (47%)	20 (22%)	0.0008*
New lesion	20	11	0.784
Recurrent lesion	14	9	
Mean days between discharge and MDT readmission	499 days (n = 34)	364 days (n = 20)	0.551

Table 5 shows the details of Charcot foot. There were 12 unique patients with Charcot foot in Cycle 1. There were 15 patients identified during Cycle 2 with Charcot foot; however, five were excluded as they were also in Cycle 1; this left 10 unique patients in Cycle 2. In terms of salient results, 90% of patients (compared to 42%) received an orthopaedic review, which correlated with more conservative management (70% vs. 75% in Cycle 1), higher healing rates (90% vs. 67%) with lower mortality at three years (20% vs. 33%) and lower recurrence (0 vs. 3 patients). Due to small sample sizes, statistical tests were not conducted.

**Table 5:** Charcot foot outcomes. Comparison of clinical outcomes in patients with Charcot neuroarthropathy between Cycle 1 (2016) and Cycle 2 (2019). Values are shown as n (%) unless otherwise stated. All percentages are expressed to two significant figures. Durations are expressed as mean days. *: Five patients were excluded because they appeared in both cycles. MDT = multidisciplinary team

	Cycle 1 (2016)	Cycle 2 (2019)
Unique patients	12	10 (5* excluded)
Orthopaedic review	5 (42%)	9 (90%)
Vascular review	6 (50%)	10 (100%)
Management: conservative	9 (75%)	7 (70%)
Management: surgical	3 (25%)	3 (30%)
Orthopaedic surgery	2 (17%)	3 (30%)
Healed	8 (67%)	9 (90%)
Not healed	3 (25%)	1 (10%)
Major amputation	1 (8%)	0
Mortality at 3 years	4 (33%)	2 (20%)
Readmitted to MDT	4 (33%)	1 (10%)
Recurrence	3	0
New lesion	1	1
Index lesion healed	8 patients	9 patients
Index lesion healing duration, days (mean)	459 days	489 days
Stayed longer in MDT	4 patients	1 patient
Mean duration in MDT after healing, days	830 days	591 days

## Discussion

This study examines the clinical outcomes before and after the introduction of a diabetic foot MDT in a district general hospital in the United Kingdom. It further reinforces findings by Meloni et al. [3] that the introduction of specialised MDT input is beneficial for these patients in terms of mortality, amputation, and, significantly in our study, ulcer healing time. Further significant improvements include the reduction in patients readmitted to MDT in 3 years and an increase in vascular reviews. The data also provided important demographic information as two separate snapshot audits, consistent with the literature in terms of baseline characteristics [12], while highlighting challenges such extra time patients spent under MDT once their index lesion healed. Overall, this study shows the benefits of a diabetic foot MDT introduction in a UK population and should provide encouragement to centres that are considering the same, given that up to a one-sixth of UK hospitals do not have a dedicated multidisciplinary diabetic foot service [8].

Earlier studies showed the improvement between MDT and our primary outcomes of interest, major amputation and mortality, presumably due to better glycaemic control, infection management, offloading, and surgical planning. Musuuza et al. [9] found a reduction in major amputations in 94% of studies, and Vuorlaakso et al. [7] reported a decrease in amputation rate by 38-82% over a range of studies and time periods. Our study showed a decrease from 11% (n = 8) to 6% (n = 5); however, this was not significant (p = 0.196). Possible reasons for non-significance include increased complexity, e.g., more patients had multiple lesions in Cycle 2, or possibly the study required a longer follow-up. For example, Rubio et al. [13] examined a six-year period before and a three-year period after MDT introduction in Madrid to establish a significant reduction in major amputations, but still found non-significance in minor amputations, a trend also supported by Wang et al. [14]. Macfarlane et al. [10], examining two cohorts in Australia in 2017 and 2019 with a one-year follow-up, found an increase in minor amputation, reflecting aggressive and earlier vascular surgery intervention, indicating higher quality care, also reported in further studies [15,16] with a “no-wait” policy. Our study reflects this, as all post-MDT patients received a vascular review and an increase in revascularisation surgery.

Similar patterns are seen with mortality. Our study showed a decrease in three-year mortality (from 39% to 29%), which was clinically meaningful, although not statistically significant (p = 0.180). A meta-analysis by Chen et al. [17] found one-year and three-year mortality for a DFU to be roughly 13.1% and 33.1%, respectively, which is in line with our MDT. Huizing et al. [11], who found statistical significance in major amputations, did not find any statistical significance in amputation-free survival (88.6% vs. 90.2% pre- and post-MDT) or total survival. The meta-analysis by Meloni et al. [3] found that only four pre-post studies had been done on mortality effects following diabetic foot MDT, where the biggest reduction was in the risk of mortality, reduced by 69 % [18,19]. Pathogenesis of mortality can occur from DFU, mainly from infection, followed by systemic sepsis; however, due to the comorbid state of diabetic foot patients, it could be due to other features such as cardiovascular events. In a one-year prospective French study, Van et al. [20] found a one-year mortality rate of 9%, with age and perfusion as risk factors for mortality. In our study, while we managed to show a lower mortality across both cycles, we did not find the cause of death or account for important comorbidity confounders. Furthermore, our absolute mortality figures may be slightly higher as we recorded patients who died within three years of a new ulcer presenting to the clinic, which often does not represent their first ulcer. Some patients may have had multiple previous ulcers over a number of years.

Encouragingly, our findings show significant improvements in ulcer healing and readmission rates. The average time to lesion healing reduced from 300 days to 244 days (p = 0.0375) alongside the improved healing rate (64% to 74%) (although not significant at p = 0.289). This shows the benefits of repeated interdisciplinary assessments controlling for neuropathy, ischaemia, and infection. According to the literature, the median time for healing varies significantly. Huizing et al. [11] found a median time for ulcer healing at 103 days (interquartile range (IQR) = 57-182) vs. 57 days (IQR = 35-113) pre- and post-MDT. Patry et al. [12] found a mean healing time of 116.8 days, with 78% of wounds healed by one year. Ince et al. [21] examined healing times for different ulcers. Neuropathic ulcers healed in 63 days (median), but neuroischaemic ulcers, which include nerve and blood vessel damage, healed in 123 days. Our longer healing time may reflect the inclusion of complex lesions such as Charcot foot, secondary osteomyelitis, and gangrene.

A persistent challenge is that many patients remain in MDT care even after their index ulcer has healed, often due to new lesions, chronic ulcers, or amputation wounds, highlighting the ongoing complexity of diabetic foot care. However, the number of people who were re-admitted due to a new or recurrent ulcer reduced after the introduction of MDT from 47% to 22% (p = 0.0008), showing the possibility of other holistic management or better wound care and compliance. Readmission into an outpatient MDT once their lesions have healed and they have been discharged has not been extensively covered in the literature. Holscher et al. [22] found inpatient readmission rates of nearly 22% within 30 days of a hospitalisation from a DFU. Similarly, Hicks et al. [23] found ulcer recurrence rates of 30% and 64% across one- and three-year follow-ups, respectively, which is higher than ours, but they examined a more morbid cohort undergoing vascular surgery. The number of days between discharge and re-admission was nearly one year, which was clinically, but not statistically, significant. Notably, we also observed an increase in patients with non-ulcer presentations and more excluded patients in Cycle 1, while Cycle 2 patients had possibly greater complexity with multiple concurrent ulcers, possibly reflective of different patient pathways.

Charcot foot outcomes were also improved, e.g., lower recurrence, higher healing rates, and less surgical intervention. Though no formal statistical testing was conducted due to the small sample size, it is notable that no Charcot cases reactivated within three years of discharge. Those who were conservatively managed had off-loading, a total contact cast (TCC), and an air-walker boot. Compared to the literature, this is similar to Griffiths et al. [24], who found treatment time with TCC in Charcot foot to vary from 4 to 12 months. In their study, Gratwohl et al. [25] found that roughly 42% of patients with Charcot foot underwent surgery after a median duration of 1.5 years, which is in line with our findings. In our study, there was an increase in orthopaedic surgery post-MDT, showing the benefit of regular orthopaedic input into the MDT.

The retrospective nature of this study presents several limitations. For example, there are multitudes of data points available in a diabetic foot cohort, representing one of the most complex patient cohorts. Variables such as ulcer characteristics, previous surgeries, and inpatient admissions were not collected. Similarly, it is possible that data were not collected over a large enough time period and did not capture the true benefits of MDT implementation. Therefore, primary outcome data, such as mortality or death, while clinically significant, were not statistically significant. Confidence intervals were also not included for associated p-values. Furthermore, this article addresses, in most part, the outpatient diabetic foot MDT service; however, many inpatients presented with urgent sepsis or surgical need, who were managed holistically but likely followed up in a different outpatient clinic, for example, vascular or orthopaedic, instead of the specialised diabetic foot clinic. This may have understated the number of our orthopaedic reviews. Regarding Charcot foot, sample sizes were small. Future randomised control trials, large cohort, or prospective studies will be useful to further evaluate the impact of MDT on diabetic and Charcot foot outcomes. It may also be useful to account for costs from an operational point of view, or to collect data from service records instead of patient files.

## Conclusions

The introduction of a one-stop multidisciplinary diabetic foot clinic at our district general hospital led to significant improvements in ulcer healing time and reductions in readmission rates, with encouraging trends towards lower amputation and mortality. These findings add to the growing evidence that integrated, specialist care can improve outcomes for people with diabetic foot disease. Wider implementation of such services across the United Kingdom may help reduce the burden of diabetic foot complications, though larger prospective studies are needed to confirm long-term benefits and cost-effectiveness.

## References

[1] Kerr M, Barron E, Chadwick P (2019). The cost of diabetic foot ulcers and amputations to the National Health Service in England. Diabet Med.

[2] McDermott K, Fang M, Boulton AJ, Selvin E, Hicks CW (2023). Etiology, epidemiology, and disparities in the burden of diabetic foot ulcers. Diabetes Care.

[3] Meloni M, Giurato L, Monge L (2024). Effect of a multidisciplinary team approach in patients with diabetic foot ulcers on major adverse limb events (MALEs): systematic review and meta-analysis for the development of the Italian guidelines for the treatment of diabetic foot syndrome. Acta Diabetol.

[4] National Institute for Health and Care Excellence (2015). Diabetic Foot Problems: Prevention and Management. London.

[5] International Working Group on the Diabetic Foot (2023). IWGDF Guidelines on the Prevention and Management of Diabetes-Related Foot Disease. Hague.

[6] Manu CA, Mustafa OG, Bates M (2014). Transformation of the multidisciplinary diabetic foot clinic into a multidisciplinary diabetic foot day unit: results from a service evaluation. Int J Low Extrem Wounds.

[7] Vuorlaakso M, Karèn V, Kiiski J, Lahtela J, Kaartinen I (2024). Multidisciplinary management of diabetic foot infection associated with improved 8-year overall survival. J Diabetes Complications.

[8] National Health Service (2020). Programme GIRFT: GIRFT Diabetes National Specialty Report. London.

[9] Musuuza J, Sutherland BL, Kurter S, Balasubramanian P, Bartels CM, Brennan MB (2020). A systematic review of multidisciplinary teams to reduce major amputations for patients with diabetic foot ulcers. J Vasc Surg.

[10] Macfarlane SM, Zhao SX, Lafrenz JO, Nagaratnam MV, Tchen A, Linton CE, Yuen L (2024). Effect of a multidisciplinary team approach on the management of diabetic foot ulcers on the Central Coast: a review of the Gosford Hospital High-Risk Foot Clinic. Int Wound J.

[11] Huizing E, Schreve MA, Kortmann W, Bakker JP, de Vries JP, Ünlü Ç (2019). The effect of a multidisciplinary outpatient team approach on outcomes in diabetic foot care: a single center study. J Cardiovasc Surg (Torino).

[12] Patry J, Tourigny A, Mercier MP, Dionne CE (2021). Outcomes and prognosis of diabetic foot ulcers treated by an interdisciplinary team in Canada. Int Wound J.

[13] Rubio JA, Aragón-Sánchez J, Jiménez S (2014). Reducing major lower extremity amputations after the introduction of a multidisciplinary team for the diabetic foot. Int J Low Extrem Wounds.

[14] Wang C, Mai L, Yang C (2016). Reducing major lower extremity amputations after the introduction of a multidisciplinary team in patient with diabetes foot ulcer. BMC Endocr Disord.

[15] Kim CH, Moon JS, Chung SM (2018). The changes of trends in the diagnosis and treatment of diabetic foot ulcer over a 10-year period: single center study. Diabetes Metab J.

[16] Plusch D, Penkala S, Dickson HG, Malone M (2015). Primary care referral to multidisciplinary high risk foot services - too few, too late. J Foot Ankle Res.

[17] Chen L, Sun S, Gao Y, Ran X (2023). Global mortality of diabetic foot ulcer: a systematic review and meta-analysis of observational studies. Diabetes Obes Metab.

[18] Ayada G, Edel Y, Burg A (2021). Multidisciplinary team led by internists improves diabetic foot ulceration outcomes a before-after retrospective study. Eur J Intern Med.

[19] Martínez-Gómez DA, Moreno-Carrillo MA, Campillo-Soto A, Carrillo-García A, Aguayo-Albasini JL (2014). Reduction in diabetic amputations over 15 years in a defined Spain population. Benefits of a critical pathway approach and multidisciplinary team work. Rev Esp Quimioter.

[20] Van GH, Amouyal C, Bourron O (2021). Diabetic foot ulcer management in a multidisciplinary foot centre: one-year healing, amputation and mortality rate. J Wound Care.

[21] Ince P, Kendrick D, Game F, Jeffcoate W (2007). The association between baseline characteristics and the outcome of foot lesions in a UK population with diabetes. Diabet Med.

[22] Holscher CM, Hicks CW, Canner JK (2018). Unplanned 30-day readmission in patients with diabetic foot wounds treated in a multidisciplinary setting. J Vasc Surg.

[23] Hicks CW, Canner JK, Mathioudakis N, Lippincott C, Sherman RL, Abularrage CJ (2020). Incidence and risk factors associated with ulcer recurrence among patients with diabetic foot ulcers treated in a multidisciplinary setting. J Surg Res.

[24] Griffiths DA, Kaminski MR (2021). Duration of total contact casting for resolution of acute Charcot foot: a retrospective cohort study. J Foot Ankle Res.

[25] Gratwohl V, Jentzsch T, Schöni M, Kaiser D, Berli MC, Böni T, Waibel FW (2022). Long-term follow-up of conservative treatment of Charcot feet. Arch Orthop Trauma Surg.

